# Diffusion of e-health innovations in ‘post-conflict’ settings: a qualitative study on the personal experiences of health workers

**DOI:** 10.1186/1478-4491-12-22

**Published:** 2014-04-23

**Authors:** Aniek Woodward, Molly Fyfe, Jibril Handuleh, Preeti Patel, Brian Godman, Andrew Leather, Alexander Finlayson

**Affiliations:** 1 King’s International Development Institute, King’s College London, Strand, London, UK; 2 King’s Centre for Global Health, King’s College London and King’s Health Partners, Weston Education Centre, London, UK; 3 London School of Hygiene & Tropical Medicine, Keppel Street, London, UK; 4 Department of Education, King’s College London, London, UK; 5 Amoud University, Boroma, Somaliland; 6 The Department of War Studies at King’s College London, The Strand Campus, London, UK; 7 Division of Clinical Pharmacology, Karolinska Institute, Karolinska University Hospital Huddinge, Stockholm, Sweden; 8 Srathclyde Institute of Pharmacy and Biomedical Sciences, Strathclyde University, Glasgow, UK; 9 Green Templeton College and Department of Primary Care, University of Oxford, Oxford, UK; 10 Medicine Africa, Oxford, UK

**Keywords:** Diffusion of innovation, E-health, Health workforce, Perceptions, Post-conflict, Liberia, West Bank and Gaza, Sierra Leone, Somaliland

## Abstract

**Background:**

Technological innovations have the potential to strengthen human resources for health and improve access and quality of care in challenging ‘post-conflict’ contexts. However, analyses on the adoption of technology for health (that is, ‘e-health’) and whether and how e-health can strengthen a health workforce in these settings have been limited so far. This study explores the personal experiences of health workers using e-health innovations in selected post-conflict situations.

**Methods:**

This study had a cross-sectional qualitative design. Telephone interviews were conducted with 12 health workers, from a variety of cadres and stages in their careers, from four post-conflict settings (Liberia, West Bank and Gaza, Sierra Leone and Somaliland) in 2012. Everett Roger’s diffusion of innovation-decision model (that is, knowledge, persuasion, decision, implementation, contemplation) guided the thematic analysis.

**Results:**

All health workers interviewed held positive perceptions of e-health, related to their beliefs that e-health can help them to access information and communicate with other health workers. However, understanding of the scope of e-health was generally limited, and often based on innovations that health workers have been introduced through by their international partners. Health workers reported a range of engagement with e-health innovations, mostly for communication (for example, email) and educational purposes (for example, online learning platforms). Poor, unreliable and unaffordable Internet was a commonly mentioned barrier to e-health use. Scaling-up existing e-health partnerships and innovations were suggested starting points to increase e-health innovation dissemination.

**Conclusions:**

Results from this study showed ICT based e-health innovations can relieve information and communication needs of health workers in post-conflict settings. However, more efforts and investments, preferably driven by healthcare workers within the post-conflict context, are needed to make e-health more widespread and sustainable. Increased awareness is necessary among health professionals, even among current e-health users, and physical and financial access barriers need to be addressed. Future e-health initiatives are likely to increase their impact if based on perceived health information needs of intended users.

## Background

Technological innovations hold potential for health systems across the globe, including those from higher and lower income settings, to improve access, quality and cost-effectiveness of healthcare provision. ‘Technology fixes’ [1] are particularly promising for the most resource and geographically constrained settings which tend to struggle the most with providing their populations with accessible and quality health services.

Low- and middle-income ‘post-conflict’ (see Table 1 for definition) countries represent some of the most precarious health systems globally with poor capacity and significant governance challenges, and are frequently less accessible to the outside world. Most post-conflict countries are prone to further outbreaks of violence, which means they are quite different from comparable low and middle-income countries that remain stable. Human resources for health shortages are most acute in post-conflict settings due to the destruction of health and educational facilities, and death or migration of large numbers of skilled health workers [2-5]. Human resources for health density significantly affects three key Millennium Development Goal (MDG) indicators, namely maternal, infant and under-five mortality rates [6]. Therefore it is not surprising that conflict-affected areas have some of the largest MDG deficits [7,8].

**Table 1:** Key terms and definitions

	
**Innovation**	‘An idea, practice, or object that is perceived as new by the individual or other unit of adoption’ [9].
**Diffusion**	‘The process by which an innovation is communicated through certain channels over time among the members of a social system’ [9].
**Post-conflict**	‘Four features characterise post-conflict situations: (1) The signing of a formal peace agreement; (2) A process of political transition, by elections, military or civilian coups; (3) Increased levels of security; (4) A perception among national and international actors that there is an opportunity for peace and recovery’ [10].
**E-health**	‘The transfer of health resources and health care by electronic means’ [11].

Besides its challenges, post-conflict reconstruction also presents opportunities to consider options for innovation and change to train, retain, and distribute human resources for health. One such innovation that holds much promise is ‘e-health’ - the use of information and communications technology to improve health (see Table 1). Post-conflict countries such as Sierra Leone, Liberia, Somaliland, and West Bank and Gaza territories have a reasonably developed telecommunications infrastructure, and therefore potential for e-health to strengthen the post-conflict workforce [12-14].

A structured literature search revealed that some studies have looked at specific e-health applications ranging across electronic patient records, tracking of disease outbreaks with mobile phones, supply chain management and the use of telemedicine in conflict-affected countries such as: [15-17]. The majority of this limited published work reflects simple programme descriptions, with little evidence on whether and how e-health can strengthen a health workforce recovering from conflict [18]. This study explored the personal experiences of health workers using e-health innovations in different post-conflict situations. Everett Roger’s diffusion of innovation model was used as the theoretical framework.

### Theoretical framework: Roger’s diffusion of innovation

There are a wide variety of models or theories available to better understand the adoption of technological innovations. We chose to focus on Everett Rogers’ diffusion of innovation theory for three reasons. First, while some models are specifically designed to analyse technology, such as the Technology Acceptance Model [19] or the Model PC Utilization [20], we thought a broader focus on innovation would be more appropriate ‘to maximize the discovery’ as research in this area is extremely limited in post-conflict settings [21]. Second, Roger’s innovation theory is grounded in sociology and therefore takes into account the socio-cultural context, such as social networks, cultural values, practices and beliefs which we believe are important for understanding technology use in post-conflict settings. Theory in this study is viewed as ‘a conceptual tool useful in making sense of a complex social reality’ [22], rather than a means to determine a causal relationship between individual knowledge, attitudes, or behaviours and e-health innovation use. For this reason, theories that intend to predict individual behaviour [23] or acceptance of technology [24] seemed less appropriate. Third, Roger’s work is, we believe, one of the most well-known and applied innovation theories since it was developed in the 1960s.

Rogers’ diffusion of innovation theory contains ‘mini’ models or theories including the attributes of innovations, the adopter categories (that is, earlier to adopters) and the innovation-decision process [9]. The authors focused on the innovation-decision model as we were seeking to gain a better understanding of the e-health adoption process and its possible challenges.

The innovation-decision model consists of five stages. The first is the knowledge stage, which according to Rogers is when an individual learns about the existence of a new innovation. In the persuasion stage, the individual ‘forms a favorable or unfavorable attitude toward the innovation’. The third stage involves an individuals’ decision to either ‘adopt or reject the innovation’ , and if put in use, followed by the implementation stage (fourth stage). The fifth and final stage involves confirmation, which is either ‘reinforcement’ or ‘reversal’ of an earlier innovation-decision [9].

### Definitions of key terms

Rogers’ definitions of ‘innovation’ and ‘diffusion’ were adopted. These and definitions of other key terms used in this paper can be found in Table 1. There is no common understanding in the literature of the term ‘post-conflict’. Macrae’s [10] definition was used because it is generally more specific than other commonly used descriptions such as: [25,26]. Authors recognise the confusing nature of the term ‘post-conflict’ as violent conflict continues to play a role after conflict settlements (that is, peace or ceasefire agreements) have occurred. The literature also lacks consensus on the definition of ‘e-health’. The authors used the World Health Organization’s definition [11] due to its frequency of use. Part of e-health is the use of mobile technology for health, so called ‘m-health’ , however, for simplicity reasons only the term e-health is used in this paper. This study explored the personal experiences of health workers using e-health innovations in different ‘post-conflict’ situations.

## Methods

### Study design and sites

This cross-sectional qualitative study focused on four countries: Sierra Leone, West Bank and Gaza, Somaliland and Liberia covering different ‘post-conflict’ situations. The four specific countries were chosen because of reasonably developed telecommunications infrastructure and ease of access to interviewee candidates. This study was conducted as part of a larger project for the Stockholm International Peace Research Institute. A brief summary of each site (background of the conflict, published research on e-health) can be found in the Additional file 1.

### Study participants and data collection

This study used semi-structured telephone interviews with health workers from a variety of backgrounds (that is, mixture of health cadres; those at different stages in their career; representing a variety of countries) to capture a wide variety of experiences. Sampling was purposive and based on maximum variation. Candidates were included if they: (1) had any experience of using e-health in a post-conflict setting; and (2) were proficient in spoken English. The minimum level experience deemed appropriate for inclusion was the use of electronic means to communicate with patients or other health workers.

Initially health workers were approached using contacts known to the co-authors. Further recruitment was helped by snowball sampling (that is, recommendations by initial participants). Most candidates were first contacted via email although text messages were used for those from Liberia (as an informant said this was better due to lack of Internet access). Once a potential interviewee agreed to be interviewed, information sheets and informed consent form were sent via email. These documents were verbally read to one interviewee who did not have an email address at the time. Interviewees were not remunerated for their time. Interviews were conducted by telephone in July and August 2012. This study was conducted under ethical approval from King’s College London, the Biomedical & Health Sciences, Dentistry, Medicine and Natural & Mathematical Sciences Research Ethics Subcommittee (reference: BDM/10/11-10).

All interviewees consented to being interviewed and for their interviews to be recorded. The duration of the interviews varied from 20 to 40 minutes. An initial topic guide was used including topics such as: definition of e-health; types and methods of e-health use; impact on clinical care; most effective type of e-health; barriers to e-health; and opportunities for e-health. Initial topic guide and questions were developed in consultation with a group of academics from King’s College London, the Karolinksa Institute, Stockholm, Sweden, and Somaliland using an existing framework for identifying barriers in healthcare [27]. Questions and probes were added if shown relevant after conducting the first interviews. All interviews were conducted in English and transcribed by the first author (AW).

### Data analysis

Qualitative thematic analysis with coding via NVivo 10 was used to analyse the data, using a deductive approach based on Roger’s innovation-decision model and the topic guide. These five stages and our topic guide formed codes for our initial coding framework that was developed together by two authors (AW, MF). This framework was applied to the first three interview transcripts. Codes were subsequently adapted and added if necessary. A final coding framework agreed upon via discussion between three authors (AW, MF, AF). There were no differing opinions about the coding framework. Codes were applied by two authors to all transcripts (AW, MF). Data were synthesised under separate headings, largely following Roger’s five stages, in the results section.

## Results

### Participants

Twelve semi-structured interviews were conducted by phone from London. Half the participants were recruited via direct contacts and half via snowballing. Table 2 summarises interviewee’ characteristics. One interviewee resided in another country at the time of the interview, although had undertaken undergraduate medical studies in Somaliland. Junior medical doctors included those who had not started or finished postgraduate training.

**Table 2:** Summary of characteristics of interviewees

	**N =12 (100 %) (%)(100%)**
**Gender**	
Male	7 (58%)
Female	5 (42%)
**Country representative from**	
West Bank & Gaza	4 (33%)
Somaliland	3 (25%)
Sierra Leone	3 (25%)
Liberia	2 (17%)
**Position**	
Junior medical doctor	3 (25%)
Senior medical doctor	2 (17%)
Health project worker	2 (17%)
Clinical officer	2 (17%)
Medical student	2 (17%)
Nursing student	1 (8%)

Results sections largely follow Roger’s five-staged innovation-decision model. In addition separate sections on preconditions and barriers, and social networks were included as these were important themes that emerged during data analysis. During the interviews various types of e-health innovations came up; brief descriptions of these can be found in Table 3.

**Table 3:** Descriptions of some types of e-health innovations, in alphabetical order

	
**Google Groups**	A means to ‘to participate in online discussions’ [28].
**HINARI Access to Research in Health Programme**	‘Provides free or very low cost online access to the major journals in biomedical and related social sciences to local, not-for-profit institutions in developing countries’ [29].
**MedicineAfrica**	‘An online health facility which enables doctors and other healthcare professionals to receive clinical support and training live from faculty and clinical supervision around the world interacting in small groups’ [30].
**OXPAL**	‘Collaborative partnership between students and doctors working at Oxford University and affiliated hospitals, and medical students at Al-Quds Medical School. Using an internet-based platform, tutors and students meet weekly to partake in real-time tutorials discussing clinical cases from hospitals in the Palestinian Territories’ [31].
**UpToDate**	‘Evidence-based clinical decision support resource authored by physicians to help healthcare practitioners make the best decisions at the point of care’ [32].

### Knowledge: understanding and perceptions of e-health

All interviewees had used some sort of e-health innovations, although two were not familiar with the term ‘e-health’ before the interview. When asked about their understanding of e-health, interviewees used terms such as ‘Internet’ , ‘technology’ , ‘communication’ , ‘electronic’ and ‘online’. Furthermore descriptions often had a positive undertone. In addition to e-health being beneficial to patients and doctors, a Somali doctor also highlighted the institutional benefits:

That is maybe solutions that may benefit patients as well as us, healthcare professionals and institutions.

Knowledge of the range of potential of e-health innovations was limited: most participants were able to mention only one or two different e-health applications, which were often the ones they had personal experience with. Many associated e-health primarily with information and communication technologies used for e-learning or communicating with internal peers. For example, a junior doctor from Somaliland understood e-health as a tool used to assist learning:

I understood that it’s some sort of distance learning through the Internet. That we can learn from the Internet.

Alongside its learning potential, a medical student from West Bank and Gaza felt e-health could facilitate inter- and intra-country communication:

I understand that this is the use of technology for medicine and like this to facilitate the connection between the countries, and the doctors, and to facilitate the communication between patients and doctors.

### Persuasion: attitudes towards and usefulness of e-health

Due to limited conception of e-health, and limited experience using e-health applications, many interviewees were not able to say what type of e-health they perceived most effective, or as another Somali doctor noted:

I think I can’t compare [MedicineAfrica] with any other because I don’t know how they work. I don’t know what they is or who they actually are.

However, a senior doctor from West Bank and Gaza with experience of using a wider variety e-health tools, thought medical journals to be most useful, because ‘this is what we call evidence-based medicine’.

All interviewees had generally favourable attitudes towards e-health. Positive attitudes towards e-health often stemmed from the capacity for electronic resources to help mitigate local resources constraints. This was described by several participants in regards to human resources for health. In places that face a severe shortage of human resources, especially mental health specialists and teachers, e-health innovations that allow for communication with remote experts were viewed positively. For example, a medical officer from Sierra Leone felt e-health could potentially increase access to interactions with mental health professionals abroad and consequently access to knowledge about this area of health:

We only have one retired psychiatrist here so for me working at the mental hospital, with not that much knowledge in psychiatry, I think it help me greatly in terms of information.

This medical officer thought the local University could also be persuaded, or in his words to ‘buy’ into this idea, using e-health to ‘create more intellect in terms of mental health’ and in raising interest within the medical profession. A health project worker from the same country also highlighted its benefits in the area of mental health:

Basically my opinion was for MedicineAfrica to be used in terms of exploring opportunities to improving mental health in Sierra Leone. We had the past 11 years war, which basically has caused a lot of trauma to people in Sierra Leone.

Besides perceiving e-health as useful in the area of mental health, this health project worker also viewed the sharing of information, such as on surgical procedures, with hospitals and surgeons in the ‘Western world’ and access to information via journals as potential ways to improve medical practice. A senior doctor from West Bank and Gaza also spoke positively about the usefulness of access to the latest information:

Oncology hematology is daily basis updated, trials being released in the medical journals. So to update myself regarding my work and my sub-specialty.

The potential of e-health to improve links between health workers within a country was another emerging theme. For example, the opportunities to share information and receive feedback from colleagues, but also to keep patient records, were reasons for a clinical officer from Liberia to be positive about Internet use in general and the non-health specific platform ‘Google Groups’ in particular. A junior doctor from Somaliland felt the health-specific platform ‘MedicineAfrica’ was a much-needed source of clinical teaching. This junior doctor explains the deficit in local clinical supervision for intern doctors:

Because in here, in Somaliland, supervision is so poor. And it’s very difficult to get someone to help you through your learning the clinical things.

Thus, desires to interact with health professionals abroad and to understand healthcare practices in other (higher income) settings were also important to promoting positive perceptions of e-health.

### Decision: reasons to adopt or reject e-health

Key motivations to adopt e-health innovations were to improve access to information and knowledge, or as one junior doctor in Somaliland put it, using e-health ‘for my clinical knowledge, for getting medical education’. Increasing confidence was an underlying reason to seek electronic information, by a Palestinian doctor:

When we have up to date knowledge we give better care for our patients and are more confident.

In some cases, participants adopted e-health in order to address an immediate challenge they were facing. For example, a lack of available textbooks was a reason for a medical officer from Sierra Leone to ‘go to the Internet’ to prepare himself for a talk on post-traumatic stress disorder. A Palestinian medical student added exam preparations as a reason to use the OXPAL e-learning platform. Through OXPAL, Palestinian students participate in live case tutorials led by Oxford based tutors. This Palestinian medical student explained how this e-health learning platform could supplement local medical education.

Ok, when I study for an exam I have a big book and many many subjects so I didn’t know what is the most important thing to concentrate on. So when we discussed the most important topics, I know what is the most important thing to know and to study and how to think about it and how to use the information in the correct way.

While some interviewees, who were generally further along in their careers, felt medical e-learning can potentially improve clinical skills, this Palestinian student thought it less useful for applied clinical activities:

But as a knowledge, a way how to think, and how to get information like that. It has helped me like that, but in the clinical work, how to work, how to deal with patients, this is I think more… more than e-health communication.

While e-health innovations for medical education may offer potential to teach knowledge and skills, it is a supplement, rather than a replacement for local clinical teaching.

Cost-related barriers were reasons given by some to reject certain e-health innovations, such as for a junior doctor from Somaliland:

MedicineAfrica is free you know, free to learn… But the other e-learning Internet or website that I have seen they actually ask money. So it’s not free. That’s why I only use MedicineAfrica.

However, high costs did not always lead to rejection of the e-health innovation as shown by the response of a Sierra Leonean medical officer: ‘Yes it [Internet] is very expensive but I do use it’.

The ability to record and share treatment plans was another reason to adopt e-health innovations. A Liberian clinical officer uses the non-health specific platform Google groups for such a purpose:

We find a case and I serve the patient and do diagnosis and treatment plan and blog it on the group. So it can be reviewed by our other colleagues and to see the level of work we are doing.

### Implementation: types of e-health used

Information and communication technologies were the most common type of e-health innovations mentioned. This includes using the web for general searches for medical information (for example, Google), health science information websites (for example, HINARI Access to Research in Health Programme) and synchronous e-learning platforms for medical education (for example, MedicineAfrica and OXPAL).

A senior doctor from West Bank and Gaza spoke specifically about the WHO HINARI Access to Research in Health intervention:

Well what I have, I have my computer in my office and I have access to some journals, medical journals. I am using the HINARI online for full-text medical journals. And sometimes I am asking my friend from Jordan to send me some papers.

This last sentence reflects another type of e-health regularly mentioned, namely the use of technology to communicate, often via email, with colleagues based in-country or abroad. A Palestinian doctor even consulted friends abroad in clinical decision-making.

Medical students from West Bank and Gaza and junior doctors from Somaliland mentioned the use of online case-based discussion platforms MedicineAfrica and OXPAL, which were often part of their medical training. The use of Google groups, as part of a mental health intervention, was an e-health innovation mentioned by Liberian interviewees.

Two interviewees also spoke about the use of mobile phones for Internet or other e-health activities, of which a medical officer from Sierra Leone stated:

During my work, not when I am seeing patients, when I want to do something else like to check my email. And then to have more information concerning particular issues especially health issues.

A Somali doctor based in Kenya mentioned the use of UpToDate (that is, a downloadable clinical database for computer or mobile phone) for the smart phone:

Then you get any consultations [inaudible] then you can check as many or sometimes you can see a case that you never had before. Then you see which the clinical symptoms and signs… I can have an access to these programmes when I am wherever.

However, this doctor believed UpToDate was not yet available in Somaliland.

### Confirmation: sustainability of e-health use

The challenge of transferring a particular e-health innovation taught and used abroad to the post-conflict setting (such as UpToDate from Kenya to Somaliland) was also highlighted by other interviewees. For example, a health project worker used ‘electronic data to search for information and articles’ while studying in the UK, however, had not used it since because he did not know ‘how to access it’ in Sierra Leone. A senior doctor faced a similar access problem:

I was working in Jordan [name of Centre] Centre and… we have an access to 380 medical journals there. Since then I started using this website, using this medical literature. But then I switched to the West Bank and I don’t have any access there to my NIH library.

However, because of the inaccessibility of a journal library, the earlier acceptance and accepted need of this type of innovation, motivated him to search for an alternative: ‘And I got myself the HINARI service and I started using it’.

Often those first exposed to an e-health innovation during health training continued using it after graduation, or as a clinical officer from Liberia puts it: ‘we still use it [Google Groups]’. This was also true for a junior doctor from Somaliland, although she did use MedicineAfrica ‘much less’ since graduation:

Now I am a second year intern so it’s much less for me. But when I was a medical student it was, I use it as weekly discussions, and I use it on a weekly basis. And for the intern, for the first year intern we use it regularly [inaudible], Wednesdays or Mondays. But now in my last year internship I didn’t use MedicineAfrica, because MedicineAfrica wasn’t working properly. But now it’s working and later we didn’t get any invitation for… but previously I would get invitations for the discussions, but in recent I don’t use it.

Thus, technical challenges, including a lack of follow-up, on part of MedicineAfrica was a barrier for continued use of this type of e-health.

### Preconditions and barriers: e-health access and costs

A common barrier for implementation and continuation of an e-health innovation was unreliable Internet access. For example, a health project worker from Sierra Leone who trialed MedicineAfrica a few weeks previously said: ‘I wasn’t able to log in at the time’.

Poor Internet access was even more problematic in rural as opposed to urban areas. A clinical officer working in rural Liberia at the time of the interview commented: ‘it [Internet use] will take you a lot of time’. Besides slowness, the Internet was sometimes not available in rural areas according to a health project worker from Sierra Leone:

Yeah in large city centres there is definitely Internet cafes but in the more rural areas people often don’t have electricity, never mind a computer to plug in.

Besides Internet unreliability and slowness, Internet costs were also a commonly mentioned barrier for e-health use. A Palestinian doctor noted:

Actually I don’t know if this is also true in the Western countries, but here they have many people using the same line. Something that the providers here use, they use to make money.

The same financial barriers applied for Internet access on mobile phones as a Sierra Leonean interviewee explains:

Yes although it [Internet access on mobile phones] is very expensive because the mobile companies here I think two of them only have Internet access.

Besides financial barriers imposed by Internet and mobile phone companies, charges applied by owners of e-health innovations were also mentioned barriers, such as for full-text journal articles (Palestinian doctor) or online e-learning tools other than MedicineAfrica (Somali doctor).

A Liberian participant also gave financial reasons for not owning a computer: ‘I cannot afford to get a computer for myself’. However, not owning a computer was not uncommon and most interviewees had computer access at their work or University, or if needed at an Internet café. Thus, computer access was felt less a barrier than Internet access, which is most clearly articulated by a Palestinian medical student: ‘No I have access to a computer, but the Internet connection it is the problem’.

On a more positive note, interviewees from different post-conflict settings had seen visible improvements in Internet access over recent years.

While most interviewees were computer-literate, a Liberian nursing student was still learning: ‘My major barrier is learning how to use a computer… I am not computer-literate. I start to learn’.

Interviewees who used e-health innovations involving clinical discussions with foreign experts (that is, MedicineAfrica and OXPAL) felt dependent on their availability. For example a medical student from West Bank and Gaza commented:

But I think it [use of OXPAL] was hard because the number of sessions that we have it can’t include all of the doctors and experts in the UK. They need to free some of their time to get to us.

Besides this dependence on international colleagues for e-health use, international networks were also crucial in the dissemination of these innovations in the first place.

### Social networks: innovation-dissemination actors

As outlined in the ‘confirmation’ phase, while some innovations lacked transferability to the post-conflict setting, experiences of work and or study in foreign institutions played an important role in making interviewees aware of ‘what’s out there’. International actors also played an important role in the introduction of e-health interventions during health training as discussed by a clinical officer from Liberia:

It was introduced in class when I was still in school. It was introduced how we can blog to that group and [inaudible] to do. So when I was still a student when I was training I was taught about the Google groups.

Interviewees also took on the role of disseminator themselves. A junior doctor from Somaliland stated: ‘I have persuaded a lot of the students to use MedicineAfrica’. A Somali doctor, at the time of the interview based in Kenya, had the aspiration to implement e-health tools such as ‘Up-to-date’ in Somaliland:

Actually it is just an idea we haven’t started yet but we communicated with other health professionals right now within Somaliland. They work there so they know how they can actually implement these programmes.

Scaling-up existing e-health partnerships and innovations were suggested starting points to increase e-health innovation-dissemination.

## Discussion

This study has provided an insight into the experiences of e-health use among a variety of health workers from different post-conflict settings. Using Roger’s innovation-decision model as a theoretical framework for an inductive qualitative analysis allowed us to disentangle what happens at each stage of the decision-making process (that is, knowledge, persuasion, decision, implementation and contemplation), thereby making it possible to understand the process of e-health adoption in post-conflict settings. To our knowledge, this is the first study to investigate the perceptions and experiences of health-workers in post-conflict settings in regards to their adoption of e-health. We are aware of the theoretical and methodological limitations of our study (see the ‘Study limitations’ section below), including the danger of generalising from this small-scale qualitative study, however, still some trends emerged.

There was generally a lack of awareness among interviewees on the scope of e-health innovations. The most common understanding of e-health among those interviewed is in line with the definition posed by Kwankam and colleagues which describes e-health in terms of ‘the improvement of health, and the use of ICTs to do so’ [33]. Although a wider range of e-health innovations, such as health informatics [34] and m-health applications [35] have been described in low- and middle-income countries, these were not frequently described in this study. Interviewees found it difficult to identify types of e-health other than those they used themselves, with two interviewees stating that they had not heard of the term ‘e-health’ previously. These results indicate that there is a scope for increased awareness of e-health even among current e-health users in post-conflict settings.

The predominance of e-health for communication and educational purposes found in this study appears related to the isolation that health workers in post-conflict studies experience. In addition to being in low-resource environments, participants in this study reported that the need and desire to interact with other health professionals as a driving motivation behind their adoption of e-health. It is difficult to retain health workers in areas where they are likely to feel professionally isolated and have limited access to information resources and training opportunities. These challenges to the distribution and retention of human resources for health in poor and fragile settings are well-documented [36-41]. Our study indicates that health-workers in post-conflict settings are responding to the challenge of isolation by adopting e-health innovations that connect them to an international network of health workers.

This study purposively selected health workers with some experience with e-health. Since e-health is still in its infancy in post-conflict settings, our interviewees are more likely ‘early’ than ‘late’ innovation adopters [9]. Thus, the awareness gap is expected to be even larger among health workers not (yet) using e-health innovations. A cross-sectional survey among 186 health professionals working at a teaching hospital in Pakistan also found limited knowledge and awareness of e-health in their sample [42]. This would suggest that more efforts are needed to diffuse e-health innovations among intended users in post-conflict contexts.

Findings from this study indicate that international partners play an important role in diffusion of e-health innovations into post-conflict states. Often these partners were from the global north (for example, UK and US). The risk here is that the global north drives e-health innovation exposure, which is likely to be less culturally appropriate and sustainable than if driven by the south [43,44]. For this reason we believe that south-south links at individual, institutional and national levels need more promotion while the north takes a more facilitating role. Even when e-health innovations are introduced in more similar environments, local needs and barriers need to be considered.

It is imperative that e-heath innovations are compatible with local needs of intended adopters [9,45,46]. Rogers suggests an ‘innovation can lead to needs, as well as vice versa’ [9]. A cross-sectional study cannot determine the direction of this relationship between innovations and needs. However, our results indicate the importance of perceived needs in decision-making on e-health use, particularly for the persuasion and decision stages. For example, a lack of up-to-date information in current working and learning environments was one of the main reasons for interviewees to consult resources available elsewhere using e-health. This information demand might be a starting point for actors involved in diffusion of e-health, bearing in mind that perceived needs are not always in line with actual needs, nor with what experts think individuals or institutions might need [9], and neither are they fixed [47].

In our study the information need was particularly severe in the area of mental health and amongst rural health workers. As a result of traumatic exposure to armed conflict and daily stressors [48], post-conflict populations often experience poor mental health outcomes. In addition, a lack of medical expertise in-country and stigma surrounding mental health made this an area of particular need for health workers in this study. Tele-psychiatry has seen success in improving access to mental health in rural and remote communities in high-income countries [49,50], even for treatment of post-traumatic stress disorder such as: [51], and in creating a platform for transcultural psychiatry between UK and Somali medical students [52]. However, more research is needed to determine the feasibility and effects of e-health interventions in post-conflict populations [53]. Based on perceived needs within our sample, future e-health initiatives and evaluations in post-conflict settings might be most beneficial for education and clinical support of mental health professionals and rural health workers, which might contribute to reducing health inequities in populations they serve.

Besides perceived needs, future e-health initiatives also need to consider what is perceived as ‘new’ among intended users. In this study, newness generally involved quite basic use of e-health such as for sending emails or Internet searches. This suggests that innovations in e-health should be considered at a local level. In other words ‘what is new locally’?

Health workers in post-conflict settings would already benefit from more sustained Internet access. Current barriers need to be addressed by making the Internet more reliable, affordable and faster. Post-conflict settings are generally resource constrained, therefore increased cooperation with and investments from private and commercial sectors, including social entrepreneurs [54], will be needed to advance e-health infrastructure. As the Global Health Workforce Alliance noted: technological innovations to strengthen a health workforce ‘rely upon an infrastructure with hardware, software, and human component’ [55].

### Study limitations

This study used purposive sampling and therefore has a selection bias towards those known to be e-health users. Future studies might benefit from a comparative design (e-health users *vs.* non-users) to explore differences and similarities between these groups. Due to project constraints there was bias towards post-conflict settings where contacts were readily available and towards participants that spoke English. Future research might want to explore e-health use in other post-conflict settings and among health workers who are less or have no proficiency in English. Project constraints allowed for interviews to be conducted by telephone but not face-to-face, meaning participants may have been more likely to give socially desirable responses [56]. Snowball sampling possibly made our sample less heterogeneous (that is, interviewees tended to recommend candidates with experience with similar e-health innovations), although the final sample contained a good mixture (that is, male and female health workers from various health cadres and stages in their careers from four different post-conflict countries). Despite its shortcomings, this study adds to the knowledge on health workers using e-health innovations in challenging settings and provides direction for future research.

## Conclusions

Positive perceptions and experiences among health workers in this study showed e-health innovations can serve information and communication needs in post-conflict settings, particularly in areas of mental and rural health. However, more effort and investment is needed to make this a widespread and sustained reality. A previous study argued for a model of e-health adoption in isolated rural areas which relied on the content (‘clicks’), existing infrastructure (‘bricks’) and techniques used for roll out (‘tricks’) [57]. Our study highlights a deficient understanding of the clicks, which may be the result of poor design and therefore limited engagement or poor marketing. It suggests intermittent access to the bricks and therefore simplicity of access being important. Lastly there is limited evidence of alignment of implementation with real user scenarios that suggest the benefit of particular role out tricks. We recommend that real end users be engaged in the strategic planning of e-health both to facilitate the adoption process and to avoid barriers, that access be targeted at existing points of user engagement with ICT rather than project specific ICT role out and lastly that diffusion be maximized by targeting the user needs which appears around communication, information and education.

## Competing interests

AF is the Director of an e-health project in Somaliland called MedicineAfrica, which aims to connect remote health workers.

## Authors’ contributions

AW collected, transcribed and analysed the data and drafted the manuscript. MF provided theoretical insights and analysed the data. AF conceived the study and design and provided overall coordination. JH, PP, BG and AL conceived the study and participated in its design. All authors reviewed the manuscript, contributed to revision of manuscript sections, and approved this version for publication.

## Supplementary Material

Additional file 1 Country profiles. Click here for file
