# Supplementary material for: Diffusion of e-health innovations in ‘post-conflict’ settings: a qualitative study on the personal experiences of health workers
Source: Hum Resour Health. 2014 Apr 23;12:22. doi: 10.1186/1478-4491-12-22 (PMC4021507; doi:10.1186/1478-4491-12-22)
Supplement: Additional file 1 — Country profiles. [file 1478-4491-12-22-S1.docx]

**Additional: Country profiles**

*West Bank and Gaza*

The region of Palestine covers contemporary Israel, the West Bank and the Gaza strip. Israel’s population of about 6.5 million covers roughly 80% of historic Palestine and the Palestinians within the region’s occupied territories number approximately 3.5 million. Guided by the nationalist ideology of Zionism after World War II, Jewish immigrants and their descendants claimed the right to settle and control some or all of Palestine [1]. Since 1948, there were several conflicts with battle-related fatality estimates highest in 1982 [2]. After a steep drop in fatalities, there was again an upsurge of violence after the 1994 peace agreement, although deaths due to the Israel-Palestine conflict reached its lowest in 2010 [2]. To date Palestine’s conflict status remains ‘ongoing’ according to the Uppsala Conflict Data Program [2], which highlights the confusing nature of a ‘post-conflict’ setting (i.e. that they are not always conflict free). In addition the recognition of Palestinian’s authority, as opposed to Israel’s, remains disputed. Previous research shows that restricted movement of Palestinians due to Israel’s checkpoints and permits negatively affects access to health services [3, 4], causing “grave and unacceptable harm to the Palestinian health system” [5]. A doctoral study indicates that e-health innovations that are of educational, clinical, and administrative nature are of particular need to the health system in West Bank and Gaza [6].

A structured search of two health databases (PubMed, Web of Science) and journals dedicated to telemedicine and e-health (e.g. *Journal for Telemedicine and Telecare*, *Telemedicine Journal and E-Health*, and the *Telemedicine Journal*) was conducted in July 2012 as part of a review of the peer-reviewed literature on e-health in fragile states for the Stockholm International Peace Research Institute. This search revealed two studies on e-health in West Bank and Gaza. One study reported on telemedicine for trauma care and found the Internet connection in this territory sufficient for telemedicine use [7]. Another described the design of a telemedicine model without further comments on adoption of or barriers to the use of this e-health innovation [8,9].

*Somaliland*

In May 1991, Somaliland unilaterally declared its independence from the rest of Somalia, which was at the time rapidly sliding into a state of anarchy without a recognized central government. Somaliland, like Palestine, has not been recognised by the international community as an independent state. Somaliland – the North Western region of Somalia – suffered greatly due to extreme bombardments by Siad Barre and his repressive regime and military forces in the 1980s. Civil war intensified between 1988-1991 with the Somali National Movement fighting for Somaliland’s independence [10]. This resulted in approximately 50,000 people killed, total destruction of its capital Hargeisa, and over half a million people were forced to flee [11]. The civil war destroyed most of its healthcare and educational facilities, and resulted in death and migration of large numbers of health workers [10, 13]. However, despite some civil unrest in 1994-1996 Somaliland has been relatively peaceful, and has even by several experts been described as “a successful case of African state-building” [such as: 41, p441]. The health sector has witnessed progress, including the opening of new health training institutions [10], however, is still struggling, particularly in providing mental health care [10, 15]. A founder of Somaliland’s Nursing & Midwifery Association suggests that “appropriate technology can play a much greater role in improving the education of health workers”, with e-learning potentially “useful for teaching post-basic specialisations like mental health or emergency skills” [10, p22].

Two country relevant peer-reviewed articles were found via a structured literature search in July 2012: one described a pilot study of an internet-based telepsychiatry [16] and another a case-based distance-learning collaboration between the UK and Somaliland [17]. These studies showed that e-health innovations have the potential to fill clinical and educational gaps in Somaliland (e.g. Finlayson et al noted that IT knowledge and Internet availability sufficed), however since both are preliminary studies further research is required.

*Liberia*

Economic decline in the 1970s resulted in dissatisfaction and unrest among Liberians, resulting in ‘brain drain’ of large numbers of health professionals [18]. Liberia’s civil war started in 1989, when Charles Taylor’s National Patriot Front of Liberia (NPLF) forces, infiltrated the country via neighbouring Côte D’Ivoire, and ended in 2003 by signing of a peace agreement [18, 19]. The war exacerbated the international migration of professionals, with almost no medical specialists left by 1990 [18]. Consequently, the depleted health workforce together with insufficient health facilities was unable to provide for Liberia’s health needs, including those internally displaced in its capital Monrovia [18]. An assessment by its new government in 1997, revealed 90% of existing health facilities were “looted, damaged, or destroyed” because of the conflict [20, p35 based on National Health Policy 2000]. However, Lee and colleagues analysis of Liberia’s 2007-2010 National Health Policy indicated “significant progress was made” since then, such as in the alignment of health actors and scale-up of health infrastructure and workforce [21]. Despite this, poor service quality, underserved rural areas, and lack of focus on population needs are prevailing challenges, with authors suggesting “innovative methods to improve long-term retention in care and bridge the rural health delivery gap” are necessary to improve treatment and care for chronic diseases such as mental health and HIV/AIDS [21].

Work by the Georgia Tech and Carter Center Collaboration shows that e-health activities are able to strengthen mental health care in Liberia [22]. However, no peer-reviewed literature was found on e-health in Liberia during a structured search in July 2012.

*Sierra Leone*

Attracted by Sierra Leone’s diamond fields, Taylor’s forces under the name of Revolutionary United Front (RUF) eventually crossed Sierra Leone’s Eastern border in March 1991. Preying on its grievances and youth unemployment, the RUF grew and conflict spread throughout the country, targeting local leaders but also civilians, with many being “brutally killed, maimed, and raped” [23, p31]. Rebel-controlled areas, such as the diamond-rich district of Kono, but also other rural areas, particularly suffered during this time. After a “succession of coups, peace accords, elections and peacekeeping missions” [23, p31] the war is commonly perceived to have ended early 2002 [23, 24]. However, 11 years of war practically destroyed Sierra Leone’s social, economic, and health infrastructure [24, 25], including most public health clinics [25]. Cities received an influx of migrants from rural areas, including health professionals, although many also sought safer pastures abroad, leaving the country with a several depleted health workforce [25–27]. This combined with poor distribution and quality of care and high out-of-pocket expenditures resulted in limited healthcare access and some of the highest maternal and child mortality rates worldwide [28–30]. Since April 2010, the Sierra Leonean’ government has introduced some initiatives to address these challenges, such as free healthcare for pregnant and lactating women and children-under-five. Consequently, financial barriers have been reduced and there has been increased health care utilization [27], although insufficient monitoring and evaluation systems, particularly in relation to drug distribution, undermine further improvements [27, 31]. Technology might play an important role here. Large investments by mobile phone companies has already helped “the integration of rural areas into the global economy” [32, p157], and the scale-up of a project using solar panels to generate Internet access has great potential to improve Internet services throughout Sierra Leone [32] including rural areas, which could help with the diffusion of e-health approaches.

No peer-reviewed articles were found on e-health in Sierra Leone during a literature search in July 2012. However, a published research protocol proposed to assess m-health – the use of mobile phones for health – on motivation and service delivery of community health workers in Sierra Leone [33].

**References**

1. Gelvin JL: *The Israel-Palestine Conflict. One Hundred Years of War*. New York: Cambridge University Press; 2005.

2. **Uppsala Conflict Data Program. UCDP Conflict Encyclopedia** [www.ucdp.uu.se/database]

3. Rytter M, Kjældgaard A, Brønnum-Hansen H, Helweg-Larsen K: **Effects of armed conflict on access to emergency health care in Palestinian West Bank: systematic collection of data in emergency departments**. *BMJ* 2006, **332**:1122–4.

4. Vitullo A, Soboh A, Oskarsson J, Atatrah T, Lafi M, Laurance T: **Barriers to the access to health services in the occupied Palestinian territory: a cohort study [abstract]**. *Lancet* 2012.

5. Shauer A, Ziv H: **Conflict and public health: report from Physicians for Human Rights-Israel.** *Lancet* 2003, **361**:1221.

6. Baroud RM: **How Ready are the Stakeholders in the Palestinian Health Care System in the Gaza Strip to Adopt e-Health?** *PhD Thesis.* University of Calgary, Department of Community Health Sciences; 2008.

7. Todder D, Matar M, Kaplan Z: **Acute-phase trauma intervention using a videoconference link circumvents compromised access to expert trauma care**. *Telemed J E Heal* 2007, **13**:65–67.

8. Zatari D, Tahboub R, Nawahda M, Aljabari T: **Design and implementation of a telemedicine system in Palestine**. *J Telemed Telecare* 1999, **5**(Suppl 1):S39–41.

9. Zatari D: **Design of a centralized telemedicine model in Palestine**. *J Telemed Telecare* 2002, **8**(Suppl 2):96–97.

10. Ismail FM: *Patience and Care. Rebuilding Nursing and Midwifery, in Somaliland*. London; 2011.

11. **Armed Conflict Database - Somalia -Historical Background** [http://www.iiss.org/publications/armed-conflict-database/]

13. Leather A, Ismail EA, Ali R, Abdi YA, Abby MH, Gulaid SA, Walhad SA, Guleid S, Ervine IM, Lowe-Lauri M, Parker M, Adams S, Datema M, Parry E: **Working together to rebuild health care in post-conflict Somaliland.** *Lancet* 2006, **368**:1119–25.

14. Renders M: **Appropriate “governance-technology”? – Somali clan elders and institutions in the making of the “Republic of Somaliland.”***African Spectr* 2007, **42**:439–459.

15. WHO: *AIMS Report on Mental Health System in Somaliland Region of Somalia*. Geneva; 2006.

16. Abdi YA, Elmi JY: **Internet based telepsychiatry: a pilot case in Somaliland**. *Med, Confl Surviv* 2011, **27**:145–150.

17. Finlayson AET, Baraco A, Cronin N, Johnson O, Little S, Nuur A, Tanasie D, Leather A: **An international, case-based, distance-learning collaboration between the UK and Somaliland using a real-time clinical education website.** *J Telemed Telecare* 2010, **16**:181–4.

18. Varpilah ST, Safer M, Frenkel E, Baba D, Massaquoi M, Barrow G: **Rebuilding human resources for health: a case study from Liberia.** *Hum Resour Health* 2011, **9**(11):doi:10.1186/1478-4491-9-11.

19. Petit D, Sondorp E, Mayhew S, Roura M, Roberts B: **Implementing a Basic Package of Health Services in post-conflict Liberia: perceptions of key stakeholders.** *Soc Sci Med* 2013, **78**:42–9.

20. WHO: *Liberia. Health Situation Analysis Final Report. July 2002 - November 2003*. Geneva; 2003.

21. Lee PT, Kruse GR, Chan BT, Massaquoi MB, Panjabi RR, Dahn BT, Gwenigale WT: **An analysis of Liberia’s 2007 national health policy: lessons for health systems strengthening and chronic disease care in poor, post-conflict countries.** *Global Health* 2011, **7**(37):doi:10.1186/1744-8603-7-37.

22. **Georgia Tech and the Carter Center’s Innovative Collaboration for Mental Health in Liberia** [http://psychcentral.com/blog/archives/2011/12/13/georgia-tech-and-the-carter-centers-innovative-collaboration-for-mental-health-in-liberia/]

23. Vincent JBM: **A Village-Up View of Sierra Leone’s Civil War and Reconstruction**. *IDS Bull* 2013, **44**:30–43.

24. Geberie L: *A Dirty War in West Africa. The RUF and the Destruction of Sierra Leone*. Indiana: Indiana University Press; 2005.

25. Desai A: **Sierra Leone’s long recovery from the scars of war**. *Bull World Health Organ* 2010, **88**:725–726.

26. Wurie AT: **Education reconstruction in post-conflict Sierra Leone**. *Commonw Educ Partnerships 2007* 2007:61–64.

27. Maxmen A: **Sierra Leone’s free health-care initiative: work in progress**. *Lancet* 2013, **381**:191–192.

28. Wakabi W: **Mothers and infants to get free health care in Sierra Leone**. *Lancet* 2010, **375**:882.

29. MoHS: *National Health Sector Strategic Plan 2010-2015*. Freetown; 2009.

30. Oyerinde K, Harding Y, Amara P, Kanu R, Shoo R, Daoh K: **The status of maternal and newborn care services in Sierra Leone 8 years after ceasefire.** *Int J Gynaecol Obstet* 2011, **114**:168–73.

31. Amnesty International: *At a Crossroads. Sierra Leone’s Free Health Care Policy*. London; 2011.

32. The Republic of Sierra Leone: *An Agenda for Change. Second Poverty Reduction Strategy (PRSP II)*. Freetown; 2012. [http://unipsil.unmissions.org/portals/unipsil/media/publications/agenda_for_change.pdf]

33. Vallières F, McAuliffe E, Palmer I, Magbity E, Bangura A: **Supporting & Strengthening maternal, neonatal, and child health services using mobile phones in Sierra Leone: A Research Protocol**. *Harvard Africa Policy J* 2013, **8**:46–51.
